# Supplementary material for: Patterns of Intron Gain and Loss in Fungi
Source: PLoS Biol. 2004 Nov 30;2(12):e422. doi: 10.1371/journal.pbio.0020422 (PMC532390; doi:10.1371/journal.pbio.0020422)
Supplement: Table S1 — Also available at http://genes.mit.edu/NielsenEtAl/. (4.3 MB ZIP). [file pbio.0020422.st001.zip › NielsenEtAl/html/369.html]

AN4775.1.NCU02650.1.MG02608.1.FG09432.1


```
 CLUSTAL W (1.82) Multiple Sequence Alignments - Introns Inserted


Sequence 1: NCU02650.1	494 aa
Sequence 2: FG09432.1	483 aa
Sequence 3: MG02608.1	507 aa
Sequence 4: AN4775.1	487 aa
Alignment Length: 512 aa
Number Identitical Residues: 304 aa
Alignment Score (without introns) 13827


MG02608.1 	MADAAFKPEKDFSKEADKLIPEAEKLAK~S-DIQGAIEKLSVLEKQAR-~-Q0ASDLATT
NCU02650.1	MAEPAFKPEKDFSKEVDKLLPEAEQLAK0T-DIQAAIEKLSVLEKQTR-~-Q0ASDLAST
FG09432.1 	MSDGVLKPEKDFSKEVDQQLPEAEKLAA0SNNLQGAIEKLAALEKQTRQ0DQ~ASDLAST
AN4775.1  	-MSGVLKPEKDFSKDADKLIPEAEQLAK0T-DVQGAIDKLLLLEKQAR-~-Q0SSDLPTT
          	  . .:********:.*: :****:**  : ::*.**:**  ****:*   * :***.:*

MG02608.1 	SRVLVAIVTICKDEGDWSLLNDQVLVLSKKHSQLKQAITKMVQTVVTFLDSTPNLETKLS
NCU02650.1	SRILVAIVTICKNAGDWSLLNDQTLVLSKKHGQLKQAITKMVQTVIDFLPETPNLETKLS
FG09432.1 	SRVLIAIVTLCKNAGDWSLLNDQTLVMSKKHSQLKQAITKMVQTVMGFLDDTPDLQTKLS
AN4775.1  	SRLLVTIVTISKNTGDWNLLNDQVLLLSKKHGQLKQAISRMVQTVMSFLDETPNMETKLS
          	**:*::***:.*: ***.*****.*::****.******::*****: ** .**:::****

MG02608.1 	VIETLRTVTEGK~IFVEVERARVTKILSDIKKEQGDLKAATDILCELQVETFGSMERREK
NCU02650.1	VIETLRTVTEGK~IFLEVERARVTKILSDIKKEQGDLKAATDILCELQVETFGSMDRREK
FG09432.1 	VIETLRTVTEGK~IFVEVERARVTKILSDIKKKQGDLKSATEILCELQVETFGSMDRREK
AN4775.1  	VIQTLRTVTEGK0IFVEVERARVTRILSQIKKSQGDLNAAADILCELQVETFGSMTRREK
          	**:********* **:********:***:***.****::*::************* ****

MG02608.1 	TQFLLDQVALCIESGDWTQAGILSRKISTKYLARKPKKTPEQLEKEKQEREK--KAKKGD
NCU02650.1	TEFILAQVALCIESKDWTQAGILSRKISTKYLARRPKKTQEQLDKEQKDREA--KKARGE
FG09432.1 	TEFILAQVELCIESEDWTQAAILGRKISTRYLSRKPKKTAEQIEKEQKEREK--KIARGE
AN4775.1  	TEFILEQVALCIERGDWTQATVLSRKINKRYFARKPKKSAEEIEKLKKEAEEREKTRAPD
          	*:*:* ** ****  ***** :*.***..:*::*:***: *:::* ::: *  .*    :

MG02608.1 	VVPEVEVDDTTDLKLRYYEQQVILSQHEDKYLDVCKHYRQVLDTEAVEEDPAKLRA0VLQ
NCU02650.1	EVSEEKEDDVTDLKLRYYEQQIILAKHDSRYLDVCKYYRQVLDTEAVEEDPAKLQA0VLQ
FG09432.1 	EVPEEKEDDTTDLKLRYYEQQIILAKHEEKYLDVCKHYRQVLDTEAVEEDPAKLRP0VLQ
AN4775.1  	EAPMEVDDDVTDLKLRYYEQQIILANHDYKYLDVCKHYREVLDTDSVQENPEQLRA0VLA
          	 ..    **.***********:**::*: :******:**:****::*:*:* :*:. ** 

MG02608.1 	RVIYFIILSPHDNEQHDLLHRIHRDTRNSQIQLDAELLKLFTVHELMRWPEVAKTFGPHL
NCU02650.1	RIIYFIILAPHDNEQHDLLHRIHKDPRIAQVPQDAELLKLFTVHELMRWPEVSKIFGPHL
FG09432.1 	RIIYFVILAPYDNEQHDLLHRIHKDTRNSEVPAEAELLRLFTVHELMRWPEISKRFGPHL
AN4775.1  	RIVYYIVLSPYDNEQSDLLHRIQQDTRLSAVPVESRLVKLFTVPELMRWPIVAEQFGPHL
          	*::*:::*:*:**** ******::*.* : :  ::.*::**** ****** ::: *****

MG02608.1 	CSTDVFSNQPPPSASSNTTPSAATDKVVKPHKRWEDLRKRVIEHNVRVIARYYTRIRMDR
NCU02650.1	CSTEIFDSEPNQSADD------------KAFQRWQDLRKRVIEHNVRVVAKYYTRIRVDR
FG09432.1 	CSTDVFDVQPGQSSDD------------KAHKRWQDLRKRVIEHNVRVVAKYYTRIQMSR
AN4775.1  	CNTDVFSPKPSQSAED------------RPYRRWQDLRKRVIEHNVRVVAKYYTRIEMGR
          	*.*::*. :*  *:..            :..:**:*************:*:*****.:.*

MG02608.1 	LTELLDLAEEETEKYISDLVTSKTVYAKIDRPARIVNFAKPRDADDVLNEWSSNMKSLLG
NCU02650.1	LTQLLDLTEDETEKYISELVTSKTVYAKIDRPARIVSFAKPRDADDILNEWSFNMKSLLG
FG09432.1 	LTQLLDLTEDETEKYISELVTSKTVYAKIDRPARIVNFAKPRDADDVLNEWSHNMKSLLG
AN4775.1  	LTQLLDLTEEETEKYISELVTSKTIYAKIDRPARLVNFAKPRDADDVLNEWSSDMRSLLG
          	**:****:*:*******:******:*********:*.*********:***** :*:****

MG02608.1 	LLERIDHLITKEEMMANIQPGAKGKGQGEKAPSTRVK
NCU02650.1	HLERIDHLITKEEMMARIQPGAKSSKKSSKEKAAAR-
FG09432.1 	LLERIDHLITKEEMMARIQP-AK--------------
AN4775.1  	LLERIDHLITKEEMMARILPTREKAKAR---------
          	 ***************.* *  :
```
